# Supplementary material for: Comparison between self-reported and pedometer-measured physical activity in Vietnamese adolescents: A reliability and agreement study
Source: PLOS Glob Public Health. 2025 Jun 6;5(6):e0004725. doi: 10.1371/journal.pgph.0004725 (PMC12143546; doi:10.1371/journal.pgph.0004725)
Supplement: S1 Checklist — (DOCX) [file pgph.0004725.s001.docx]

S1. GRRAS checklist for reporting of studies of reliability and agreement.

| **Section** | **Item #** | **Checklist item** | **Reported on page #** |
| --- | --- | --- | --- |
| Title/Abstract | 1 | Identify in title or abstract that interrater/intrarater  reliability or agreement was investigated. | Pages 1-2 |
| Introduction | 2 | Name and describe the diagnostic or measurement device of interest explicitly. | Pages 3-5 |
|  | 3 | Specify the subject population of interest. | Pages 4-5 |
|  | 4 | Specify the rater population of interest (if applicable). | Not applicable |
|  | 5 | Describe what is already known about reliability and  agreement and provide a rationale for the study (if applicable). | Pages 3-5 |
| Methods | 6 | Explain how the sample size was chosen. State the determined number of raters, subjects/objects, and replicate observations. | Pages 7-8 |
|  | 7 | Describe the sampling method. | Pages 7-8 |
|  | 8 | Describe the measurement/rating process (e.g. time interval between repeated measurements, availability  of clinical information, blinding). | Pages 5-11 |
|  | 9 | State whether measurements/ratings were conducted independently. | Page 10 |
|  | 10 | Describe the statistical analysis. | Page 12 |
| Results | 11 | State the actual number of raters and subjects/objects  which were included and the number of replicate observations which were conducted. | Page 13 |
|  | 12 | Describe the sample characteristics of raters and  subjects (e.g. training, experience). | Pages 13-14 (Table 1)  Page 17 (Table 4) |
|  | 13 | Report estimates of reliability and agreement including measures of statistical uncertainty. | Pages 14-16 (Tables 2 and 3)  Pages 17-18 (Table 5) |
| Discussion | 14 | Discuss the practical relevance of results. | Pages 19-21 |
| Auxiliary material | 15 | Provide detailed results if possible (e.g. online). | S1 Data file |

Version based on Table I in: Kottner J, Audigé L, Brorson S, Donner A, Gajeweski BJ, Hróbjartsson A, Robersts C, Shoukri M, Streiner DL. Guidelines for reporting reliability and agreement studies (GRRAS) were proposed. J Clin Epidemiol. 2011;64(1):96-106. Available from: <https://www.equator-network.org/reporting-guidelines/guidelines-for-reporting-reliability-and-agreement-studies-grras-were-proposed/>
