# Supplementary material for: Comparison between self-reported and pedometer-measured physical activity in Vietnamese adolescents: A reliability and agreement study
Source: PLOS Glob Public Health. 2025 Jun 6;5(6):e0004725. doi: 10.1371/journal.pgph.0004725 (PMC12143546; doi:10.1371/journal.pgph.0004725)
Supplement: Attachment — Submitted filename: Reviewers comments on PGPH-D-25-00321.docx [file pgph.0004725.s003.docx]

Reviewers comments on PGPH-D-25-00321 Comparison between self-reported and pedometer-measured physical activity in Vietnamese adolescents: A reliability and agreement study.

Thank you for allowing me to review this paper. This paper is well written and well organised. There is always value in considering fit and cultural adaptations in questionnaire measurement instruments generally and I commend you on the care and attention to detail that you have undertaken in your work.

I have a number of questions that I would like you to consider and address in your manuscript.

1. Explain which ‘expert groups’ are you referring to in the Introduction (page 4, line 62), this should include references to support.
2. Is there an effect of the 3 month time period March-May that you chose for data collection both in terms of seasonality of weather (e.g., inclement weather affecting physical activity; PA) and also in terms of the school year (e.g., academic activities)?
3. Why one school only? Please describe this school more thoroughly for an international audience. For example, is it placed in an area of high/low socio-economic deprivation/affluence, is it a public or a private school? These factors can contribute to physical activity generally and especially in terms of children.
4. The PA programme that is described in the school – is this a normal programme or enhanced as a result of the research project taking place in the school?
5. Did you do a power calculation to determine the necessary sample size for the project?
6. Greater justification is needed for not using an accelerometer (which would consider intensity, duration and type of PA) and for pedometer use.
7. Did the children have access to their step count at the end of each day? A procedure point notes that the children reset the pedometer themselves. This would suggest that they could also see their daily steps. If this is the case, please comment on how you believe this could have enhanced their ability to then more accurately report their step count in the self-reported survey? Were any attempts made to ‘blind’ the children to their objectively measured daily step count?
