# Supplementary material for: Comparison between self-reported and pedometer-measured physical activity in Vietnamese adolescents: A reliability and agreement study
Source: PLOS Glob Public Health. 2025 Jun 6;5(6):e0004725. doi: 10.1371/journal.pgph.0004725 (PMC12143546; doi:10.1371/journal.pgph.0004725)
Supplement: Attachment — Submitted filename: Response to Reviewers.docx [file pgph.0004725.s005.docx]

Manuscript Title: Comparison between self-reported and pedometer-measured physical activity in Vietnamese adolescents: A reliability and agreement study

Manuscript Number: PGPH-D-25-00321

**Dear Editors-in-Chief, the PLOS Global Public Health**

Thank you for the opportunity to submit a revised version of our manuscript titled “Comparison between self-reported and pedometer-measured physical activity in Vietnamese adolescents: A reliability and agreement study” to PLOS Global Public Health. We truly appreciate the time and effort you and the reviewers have spent reviewing and providing valuable feedback. We are grateful to the reviewers for their thoughtful comments, which have helped improve our manuscript. We have revised the manuscript to address most of their suggestions, and these changes are highlighted for your reference. Below is our point-by-point Response: to the reviewers’ comments and concerns.

**COMMENTS FROM REVIEWER 1**

**Reviewer #1:** Thank you for allowing me to review this paper. This paper is well written and well organised. There is always value in considering fit and cultural adaptations in questionnaire measurement instruments generally and I commend you on the care and attention to detail that you have undertaken in your work.
I have a number of questions that I would like you to consider and address in your manuscript.

**COMMENT 1. Explain which ‘expert groups’ are you referring to in the Introduction (page 4, line 62), this should include references to support.**

RESPONSE: We added an explanation and cited a reference *“As part of this assessment, five international experts in PA measurement were invited to critically appraise a shortlist of 20 activity-based mearures. The evaluation process involved a detailed examination of key psychometric properties, such as reliability, validity, and ease of administration. Based on expert consensus, the PAQ-C was identified as one of the three measures that received substantial support, demonstrating robust measurement properties and practical applicability for assessing PA in youth populations [13]” (Page 4, Lines 60-66)*.

**COMMENT** **2. Is there an effect of the 3 month time period March-May that you chose for data collection both in terms of seasonality of weather (e.g., inclement weather affecting physical activity; PA) and also in terms of the school year (e.g., academic activities)?**

RESPONSE: Ho Chi Minh City has a stable climate year-round, with minimal extreme weather events such as storms, heavy rain, or flooding that could significantly impact outdoor activities. These months are part of the academic year when students typically maintain regular schedules, allowing for consistent data collection. Major disruptions like exams or holidays are less likely to interfere with the study. As a result, students' engagement in PA remains relatively consistent regardless of seasonal variations. We added the information in the method:

*“This data collection period was chosen as it represents a stable climate year-round in Ho Chi Minh City, with minimal extreme weather events that could significantly impact outdoor activities. Moreover, this timeframe aligned with the second semester, when students followed a structured academic schedule with regular physical education classes and recess. The majority of data was gathered before final examinations to mitigate potential declines in PA due to increased academic workload” (Page 6, Lines 103-109).*

**COMMENT 3. Why one school only? Please describe this school more thoroughly for an international audience. For example, is it placed in an area of high/low socio-economic deprivation/affluence, is it a public or a private school? These factors can contribute to physical activity generally and especially in terms of children.**

RESPONSE: We appreciate the reviewer's suggestion to clarify our rationale for selecting one school. This school was selected due to the feasibility of data collection within the resource limitations of our study. Concentrating on one school ensured that we could maintain the precision and accuracy of data collection while efficiently managing the logistical challenges associated with the study. We provided detailed description of the study location:

*“Ho Chi Minh City, the economic center of southern Vietnam, is a densely populated metropolis. Compared to other regions in Vietnam, the city has a higher prevalence of childhood overweight and obesity, likely influenced by urban lifestyles, dietary patterns, and reduced PA levels [6]” (Page 6, Lines 100-103).*

*“Go Vap District is one of the most rapidly urbanizing districts in the city and the second most populous, with 602,180 inhabitants [20]. Nguyen Du Secondary School, a public institution in Go Vap District, was selected for this study due to its large class sizes, and students were willing to cooperate with their physical education teachers who received pedometer training. The school enrolled 1,951 students across four grade levels (552 sixth grade students), with class sizes ranging from 45 to 49 students per class” (Page 6, Lines 110-115).*

**COMMENT 4. The PA programme that is described in the school – is this a normal programme or enhanced as a result of the research project taking place in the school?**

RESPONSE: The PA program at Nguyen Du Secondary School is a part of the curriculum outlined by the Vietnamese Ministry of Education and Training. This curriculum includes structured physical education courses integrated into the academic years. We revised a paragraph in Methods to make it clearer:

*“The school follows the curriculum set by the Vietnamese Ministry of Education and Training. The curriculum includes two physical education classes per week and a sports activity session every Saturday morning. The school also offers extracurricular sports clubs, like soccer and basketball, and organizes sports competitions twice a year. (Page 6, Lines 115-119).*

**COMMENT 5. Did you do a power calculation to determine the necessary sample size for the project?**

RESPONSE: We thank the reviewer for this insightful comment. We recruited all students of 6-grade classes of Nguyen Du high school. As requested by the reviewer, we calculated the sample size each study phase of the study and added the information to the method section as below:

*“In the first phase, a pilot study was conducted with a randomly selected sample of 35 students from one class to test the measurement tools. This sample size was based on previous validity and reliability studies, which recommend that 18-50 participants are typically sufficient for estimating Intraclass Correlation Coefficients (ICCs) to assess the validity between two methods [26].* *For the second phase, sample size calculations followed the formula for prevalence studies by Naing et al. [28], with a design effect (deff) of 1.5 to adjust for clustering [29]. The prevalence of low physically active was 0.24 [30]. Using a 95% confidence level (Z = 1.96) and a precision (d) of 0.05. The calculated sample size was 422. All 517 sixth-grade students from 11 remaining classes were invited to participate in the cross-sectional survey, with each class serving as a cluster unit” (Page 8, Lines 140-153).*

**COMMENT 6. Greater justification is needed for not using an accelerometer (which would consider intensity, duration and type of PA) and for pedometer use.**

RESPONSE: We agreed with the reviewer that accelerometer can collect more comprehensive data for PA study. However, it is more expensive and less accessible within our limited resources. The pedometer is simpler to use and more suitable to high-school students.

*“Although accelerometers can collect more comprehensive data such as intensity, duration and type of PA, their high cost and technical complexity make it less accessible in resource-limited countries like Vietnam. Pedometers are a cheaper and easier to use, making them more practical for larger studies and helping participants use them more effectively [16]. While pedometers do not capture PA intensity or duration, they accurately measure step counts and have demonstrated strong correlations with uniaxial accelerometers and moderate-to-vigorous PA [17, 18]. Moreover, the steps/day metric is considered a valid indicator of habitual PA [19]. Despite their limitations, pedometers are recognized as reliable and accurate tools for assessing PA in children and adolescents [7]” (Pages 4-5, Lines 73-81).*

**COMMENT 7. Did the children have access to their step count at the end of each day? A procedure point notes that the children reset the pedometer themselves. This would suggest that they could also see their daily steps. If this is the case, please comment on how you believe this could have enhanced their ability to then more accurately report their step count in the self-reported survey? Were any attempts made to ‘blind’ the children to their objectively measured daily step count?**

RESPONSE: We appreciate the constructive comment from the reviewer. The step-counting procedure required children to access their step count at the end of each day, as they were responsible for recording it in a daily activity log before resetting the pedometer the following morning. While we could not completely blind students to their step count due to the nature of unsealed pedometers, we required students to limit their attention to the step count display and not to modify their PA based on the numbers shown. One researcher conducted daily classroom visits to remind students to wear the pedometer consistently and to collect the previous day’s recorded step counts. Any inconsistencies or abnormal step count data were carefully reviewed and either verified at that time. We revised the paragraph for better clarity.

*“Although some concerns exist regarding measurement reactivity when students see their daily step totals, previous studies have indicated that this does not significant threaten measurement accuracy [42, 43].* *To reduce potential bias, students were first allowed to shake the pedometer to see how it responded. Afterward, they were told not to repeat this behavior in the following days. This helps lower their excitement and curiosity [44].* *In addition, students were required not to focus on the step count display or modify their PA based on the numbers shown. Monitoring lasted for seven days, during which researchers visited each class every morning to remind students to wear the pedometer, collect diary sheets, and check recorded step counts for inconsistencies. Any abnormal step count data were carefully examined and either verified at that time” (Pages 10-11, Lines 204-214).*

**COMMENTS FROM REVIEWER 2**

**Reviewer #2:** Comments

**COMMENT 1. Questionnaire: What is the content validity index of the questionnaire?**

RESPONSE: We thank the reviewer for this insightful comment regarding the assessment of content validity for the questionnaire. Although the Vietnamese version of the PAQ-C underwent a translation and cultural adaptation process, including expert review and pilot testing, we acknowledge that a formal assessment of the content validity index was not conducted in this study. This was primarily due to the study’s focus on evaluating reliability and comparability with objective measures of physical activity, as well as time and resource constraints during the instrument development phase. We recognize this omission as a limitation and have suggested directions for future research in the Discussion section as follows:

*“Furthermore, the absence of a comprehensive content validation process may have introduced potential biases, thereby limiting the generalizability and practical applicability of the PAQ-C. Future research should employ standardized procedures to formally evaluate the content validity of the instrument, including the quantitative assessment of the relevance and clarity of each questionnaire item” (Page 21, Lines 371-375)*

**COMMENT 2. What is the brand of the pedometer? What is its validity and reliability?**

RESPONSE: The pedometer used in this study was the ProForm SP-50 (Hefei Merrybody Sports Co., Ltd., Anhui, China), an unsealed device that senses body motion and counts steps in real time, with an accuracy of approximately ±10%. It was employed to assess children’s physical activity and has demonstrated acceptable validity when compared with the Physical Activity Questionnaire for Children (PAQ-C). However, to avoid any misunderstanding regarding potential commercial intent, we decided not to include the brand name of the device in the manuscript. Instead, we revised the manuscript to provide more details about the measurement tool as follows:

*“An unsealed commercial pedometer with an accuracy of approximately ±10% was used to assess PA of students. This small device senses body motion, counts steps in real time, and has demonstrated acceptable validity with the PAQ-C in adolescents [40]” (Page 10, Lines 197-199)*

**COMMENT 3. How did you ensure that students did not manipulate the pedometer results, such as shaking it?**

RESPONSE: On the first day, when the pedometers were distributed to the students, they were allowed to shake the device to observe how it worked. After this, they were explicitly instructed not to repeat this behavior in the following days to ensure accurate step count recordings. This method helped reduce their initial excitement and curiosity. Students were also advised not to focus on the step count display or alter their physical activity based on it. One researcher visited classrooms daily to monitor compliance and collect step count data, flagging any unusual activity levels for further review. We revised the manuscript to clarify these procedures as follows:

*“To reduce potential bias, students were first allowed to shake the pedometer to see how it responded. Afterward, they were told not to repeat this behavior in the following days. This helps lower their excitement and curiosity [44]. In addition, students were required not to focus on the step count display or modify their PA based on the numbers shown. Monitoring lasted for seven days, during which researchers visited each class every morning to remind students to wear the pedometer, collect diary sheets, and check recorded step counts for inconsistencies. Any abnormal step count data were carefully examined and either verified at that time” (Pages 10-11, Lines 205-212).*

**COMMENT 4. Why were results less than 1000 and more than 30,000 per day excluded? Please cite a reference for this.**

RESPONSE: We excluded values less than 1,000 and more than 30,000 steps per day based on established criteria from previous studies to ensure data validity and reliability. In the manuscript, we noted that these cut-offs were selected to align with hypothetical scenarios of the “least active child” and “most active child”. These values accounted for only 1% of the data points, making them a practical and reasonable objective criterion. Moreover, rounding to the nearest 1,000 steps provides easier values to remember for use in future studies. We revised the following information to address this concern:

*"Values less than 1,000 steps/day or greater than 30,000 steps/day were considered invalid and excluded, following established criteria from previous studies [44-46]. These cut-off points were chosen based on their alignment with hypothetical scenarios of the “least active child” and “most active child”, and represented 1% of the data points, ensuring a practical and reasonable objective criterion. Additionally, rounding the lower limit to the nearest 1,000 steps provides values that are easier to remember for data analysis purposes [44]" (Page 11, Lines 212-218).*

**COMMENT 5. Please add sample size calculations for Phase 1 and Phase 2.**

RESPONSE: We added the sample size calculations for both Phase 1 and Phase 2 as follows:

*“In the first phase, a pilot study was conducted with a randomly selected sample of 35 students from one class to test the measurement tools. This sample size was based on previous validity and reliability studies, which recommend that 18-50 participants are typically sufficient for estimating Intraclass Correlation Coefficients (ICCs) to assess the validity between two methods [26]” (Pages 7-8, Lines 140-144).*

*“For the second phase, sample size calculations followed the formula for prevalence studies by Naing et al. [28], with a design effect (deff) of 1.5 to adjust for clustering [29]. The prevalence of low physically active was 0.24 [30]. Using a 95% confidence level (Z = 1.96) and a precision (d) of 0.05. The calculated sample size was 422. All 517 sixth-grade students from 11 remaining classes were invited to participate in the cross-sectional survey, with each class serving as a cluster unit” (Page 8, Lines 145-153).*

**COMMENT 6. Define sufficient and insufficient physical activity in the PAQ-C and pedometer sections**

RESPONSE: We added definitions for sufficient and insufficient physical activity in both the PAQ-C and Pedometer subsections as follows:

*“A total PAQ-C score of ≤2.75 indicates insufficient activity, whereas a score of >2.75 represents sufficient activity [36]” (Page 9, Lines 175-177).*

*“PA levels were classified according to Rowe et al., with <10,000 steps/day indicating insufficient activity and ≥10,000 steps/day considered sufficient activity [44]” (Page 11, Lines 225-227).*

**STATISTICAL ANALYSIS
COMMENT 1. Lines 185 -186 should be part of the method.**

RESPONSE: We moved them to the method section.

**COMMENT 2. Line 199: Cohen’s Kappa test was calculated to measure the level of agreement between the two methods. Can you give classification of interpreting results.**

RESPONSE: We added the following sentence in the Statistical Analysis to clarify the interpretation of Cohen’s Kappa results:

*“The strength of agreement was interpreted as follows: ≤0.20 as none to slight, 0.21–0.40 as fair, 0.41–0.60 as moderate, 0.61–0.80 as substantial, and 0.81–1.00 as almost perfect agreement [52]” (Page 12, Lines 246-248).*

**ETHICAL CONSIDERATIONS**
**COMMENT 1. This should be part of the method section.**

RESPONSE: We moved the ethical consideration to method section.

**COMMENT 2. Was the assent form obtained from children?**
RESPONSE: Written informed consents were collected from both students and their legal guardians. We revised the sentence “*Written informed consent was obtained from parents or legal guardians, and assent forms were collected from student before data was collected” (Page 5, Lines 92-94).*

**RESULTS
COMMENT 1. Please include a flow diagram.**

RESPONSE: We added a flow diagram in the Results section.

*Fig 1. The flow diagram of the study (Page 13, Lines 259-260)*

**COMMENT 2. Kindly include gender results in the first paragraph of the result section**

RESPONSE: As the reviewer’s request, we added the gender results: *“Table 1 displays the characteristics of the study participants across the two phases. The mean age was 11.8 ± 0.2 years in the first sample (N=35) and 11.8 ± 0.3 years in the second sample (N=475). The gender distribution was comparable between the two samples, with 51.4% boys and 48.6% girls in the first sample and 50.7% boys and 49.3% girls in the second sample” (Page 13, Lines 261-264).*

In addition to addressing the reviewers' comments, we have carefully reviewed the manuscript to correct any spelling or grammatical errors. We look forward to your response regarding our submission and remain available to address any additional questions or comments you may have.
